# Antimicrobial Resistance of Neisseria Gonorrhoeae in a Newly Implemented Surveillance Program in Uganda: Surveillance Report

**DOI:** 10.2196/17009

**Published:** 2020-06-10

**Authors:** Meklit Workneh, Matthew M Hamill, Francis Kakooza, Emmanuel Mande, Jessica Wagner, Olive Mbabazi, Rodney Mugasha, Henry Kajumbula, Richard Walwema, Jonathan Zenilman, Patrick Musinguzi, Peter Kyambadde, Mohammed Lamorde, Yukari C Manabe

**Affiliations:** 1 Division of Infectious Diseases Johns Hopkins School of Medicine Baltimore, MD United States; 2 Infectious Disease Institute Kampala Uganda; 3 Bayview Pediatric Unit Johns Hopkins University Baltimore, MD United States; 4 AIDS Control Program, Division of Sexually Transmitted Infections Ministry of Health Kampala Uganda

**Keywords:** gonorrhea, antimicrobial resistance, surveillance, Uganda, STD, STI, sexually transmitted, Neisseria Gonorrhoeae, antibiotic resistance, EGASP

## Abstract

**Background:**

Neisseria gonorrhoeae (commonly known as gonorrhea) has developed resistance to all first-line therapy in Southeast Asia. East Africa has historically had absent or rudimentary gonorrhea surveillance programs and, while the existence of antimicrobial-resistant gonorrhea is recognized, the extent of its resistance is largely unknown. In 2016, the World Health Organization’s Enhanced Gonococcal Antimicrobial Surveillance Program (EGASP) was initiated in Uganda to monitor resistance trends.

**Objective:**

This study characterizes gonorrhea and antibiotic resistance in a large surveillance program of men with urethral discharge syndrome from Kampala, Uganda.

**Methods:**

Men attending sentinel clinics with urethritis provided demographic information, behavior data, and a urethral swab in line with the World Health Organization’s EGASP protocols for culture, identification, and antibiotic-sensitivity testing using 2 methods—disk diffusion (Kirby-Bauer test) and Etest (BioMérieux Inc). A subset of samples underwent detailed antimicrobial resistance testing.

**Results:**

Of 639 samples collected from September 2016 to February 2018, 400 (62.6%) were culture-positive though 414 (64.8%) had microscopic evidence of gonorrhea. The mean age of the men from whom the samples were collected was 26.9 (SD 9.6) years and 7.2% (46/639) reported having HIV. There was high-level resistance to ciprofloxacin, tetracycline, and penicillin (greater than 90%) by Kirby-Bauer disk diffusion and 2.1% (4/188) had reduced azithromycin sensitivity by Etest. Of the early isolates that underwent detailed characterization, 60.3% (70/116) were culture-positive, 94% (66/69) isolates were either ciprofloxacin-resistant or ciprofloxacin-intermediate by Etest, 96% (65/68) were azithromycin-sensitive, and 96% (66/69) were gentamicin-sensitive. Resistance profiles were comparable between methods except for ceftriaxone (disk diffusion: 68/69, 99%; Etest: 67/69, 97%) and for gentamicin (disk diffusion: 2/8, 25%; Etest: 66/69, 96%) sensitivity.

**Conclusions:**

This is the first report from a systematic gonorrhea surveillance program in Uganda. Findings demonstrated resistance or increased minimum inhibitory concentration to all key antigonococcal antibiotics. There was evidence of poor antibiotic stewardship, near-universal resistance to several antibiotics, and emerging resistance to others. Individuals in the population sampled were at exceptionally high risk of STI and HIV infection requiring intervention. Ongoing surveillance efforts to develop interventions to curtail antimicrobial-resistant gonorrhea are needed.

## Introduction

*Neisseria gonorrhoeae* (also known as gonorrhea) is a common sexually transmitted infection (STI) and a major cause of morbidity. Gonorrhea has developed antimicrobial resistance to all classes of antibiotics used in its treatment. Gonorrhea can cause sequelae such as pelvic inflammatory disease with resultant ectopic pregnancy [1] and increases HIV transmission [2]. In 2016, 86.9 million of an estimated 376.4 million new, curable STIs in adults aged 15 to 49 years were attributed to gonorrhea [3]. In 2016, the global annual incidence rate of gonorrhea was estimated at 2.6% among men and 2.0% among women. In Africa, the incidence rates were 1.6% and 1.9% among men and women, respectively [3], which was an increase from 2012 [4]. In the 1970s, gonococcal plasmid- and chromosomally mediated resistance to penicillin and tetracycline emerged in Asia, and within a decade, had spread globally [5]. High-level fluoroquinolone resistance evolved in the early to mid 2000s [6]. Third-generation extended-spectrum cephalosporins are the mainstay of gonorrhea therapy in many regions; however, their minimum inhibitory concentration has been increasing since their widespread introduction as a treatment for gonorrhea. More recently, clinical gonorrhea isolates with high azithromycin minimum inhibitory concentration have been recognized and increasingly reported [5,7].

Cases of highly resistant gonorrhea have been reported in several regions [8-10] and likely represent international clonal expansion [11,12]. Sub-Saharan African gonorrhea data are minimal [13-17]; gonorrhea resistance has been documented in Uganda [18,19], but its prevalence is unknown. In 2016, the WHO initiated its Enhanced Gonococcal Antimicrobial Surveillance Program (EGASP) in Uganda to monitor patterns of resistance. The Gonococcal Antimicrobial Surveillance Program (GASP) had been in place since 1992 to monitor antimicrobial resistance worldwide with the aim of informing treatment guidelines [20]. The WHO released the Baseline Report on Sexually Transmitted Infection Surveillance [21] in 2012, at which time there were no GASP regional focal points in Africa; in contrast, in all other WHO regions, there was at least one. In addition, only 5 countries in Africa were participating in GASP at that point in time which was an inadequate response given the burden of gonococcal disease in the region [4]. WHO GASP data from 2009 to 2014 [22] which were reported from 3 sites in Africa (South Africa, Kenya, and Côte d’Ivoire) showed persistent and widespread resistance to penicillin, tetracycline, and ciprofloxacin; increasing resistance to azithromycin (greater than 5%); and emerging resistance and decreased susceptibility to extended-spectrum cephalosporins (although only 15% of countries in Africa reported extended-spectrum cephalosporin data). Data collected from 2015 to 2016 in Zimbabwe described 9.5%-30.8% ciprofloxacin resistance and 100% sensitivity to extended-spectrum cephalosporins [23]. With EGASP, the WHO aimed to address the limitations that were identified in implementing GASP [20]. EGASP protocols standardize sampling strategies, laboratory methods, demographic information, and quality assurance procedures for use in sentinel surveillance sites in selected countries. Focusing EGASP on resource-limited settings allows more detailed scrutiny of the global burden of antimicrobial resistance in areas where prevalence is high. EGASP strengthens and streamlines reporting mechanisms for specified alert values for antimicrobial resistance, thus facilitating timely responses. In Uganda, EGASP represents a collaboration between the National STI Control Program at the Ugandan Ministry of Health, the Infectious Disease Institute in Kampala, and is carried out in partnership with the Centers for Disease Control and Prevention to implement a gonorrhea surveillance program. Herein, we report initial antimicrobial resistance and epidemiological results of the gonorrhea surveillance program in Kampala, Uganda.

## Methods

### Clinical Setting

Between September 2016 and February 2018, urethral samples were collected from men at nine sentinel clinics in and around Kampala, Uganda. Clinics were selected after reviewing health management information system records at the Ministry of Health and were based upon their ability to meet several criteria. Clinics were required to have high patient volumes in order to collect a sufficient number of samples regularly, an ability to collect data in accordance with Uganda’s national STI guidelines which involved recording a patient’s full medical history and performing a clinical examination (see Multimedia Appendix 1), and the capacity to collect and maintain sample viability until transportation to the reference laboratory. In order for samples to be properly collected, trained clinical staff were required, and to ensure safe handling of samples, a system for storage until samples were collected and transported (which occurred daily) was required.

### Sample Collection and Testing

Urethral Amies swabs without charcoal (Deltalab SL) were used to consecutively collect specimens from men who presented with urethral discharge syndrome. Samples were stored at ambient temperature and atmosphere and were transported within 12 hours to the designated reference laboratory site in Kampala (Infectious Disease Institute Translational Laboratory). Samples were inoculated on modified Thayer-Martin and chocolate agar and incubated between 35°C and 36.5°C in 5% carbon dioxide. Isolation and confirmation of *N gonorrhoeae* was performed with technical assistance from the Department of Microbiology, Makerere University. Presumptive identification of *N gonorrhoeae* was based upon (1) growth of typical colonies on modified Thayer-Martin agar between 35°C and 36.5°C in 5% carbon dioxide, (2) a positive oxidase test, and (3) observation of Gram-negative, oxidase-positive diplococci in stained smears. Antimicrobial susceptibility tests using Kirby-Bauer disk diffusion were performed for the following antibiotics: penicillin, tetracycline, ciprofloxacin, cefoxitin, gentamicin, spectinomycin, ceftriaxone, and cefixime. Additional antimicrobial susceptibility tests were performed using Etest strips (BioMérieux Inc) to determine minimum inhibitory concentration for a subset of isolates for the following priority antibiotics: cefixime, ceftriaxone, cefuroxime, ciprofloxacin, azithromycin, and gentamicin on gonococcal medium base inoculated with 10^4^ colony forming units. Etest strips are expensive and difficult to source in Uganda, and therefore, were used routinely early in the program, after which disk diffusion was used routinely to determine antimicrobial susceptibility. Presumptive *N gonorrhoeae* isolates with minimum inhibitory concentrations that exceeded criteria specified by EGASP protocols (antibiotic alert value criteria: ceftriaxone, ≥0.125 µg/mL; cefixime, ≥0.25 µg/mL; azithromycin, ≥2 µg/mL; gentamicin ≥16 µg/mL) underwent additional Etest analysis according to Biochemical Test Clinical and Laboratory Standards Institute Guidelines within five working days.

### Quality Assurance and Quality Control

Due to constraints on resources, the EGASP-recommended WHO *N gonorrhoeae* K and L control strains were not used. Alternative quality measures that could be locally implemented were used. The Becton-Dickinson control strain (American Type Culture Collection; ATCC 49226) was used in the place of WHO *N gonorrhoeae* K and L strains. Control tests were carried out monthly or upon receipt of a new batch of antimicrobial susceptibility disks, Etest strips, or the introduction of a new batch of media. Isolate identification was performed to ensure consistency in methods and the zones of clearance for the control strain were compared to the reference standards for each drug.

### Data Management and Analysis

Demographic and behavior data were collected using the combined WHO–Ministry of Health Uganda data collection form, albeit the same-sex sexual activity question was removed to reduce the risk of harm to respondents [24]. Data were manually entered into Access database software (Microsoft Inc) prior to data analysis. Minor amendments were made to the questionnaire partway through the time period under review to be more realistic in expectations of the individual’s ability to recollect events and to reduce the chance of recall bias—the period for number of gonorrhea episodes and number of sex partners was reduced from 12 to 6 months. Similarly, the period for recent, previsit antibiotic use was reduced from 60 to 14 days. These differences were reflected in the different timeframes used for reporting. Antimicrobial susceptibility data were exported to WHONet, a WHO information system for managing, analyzing, and reporting antimicrobial resistance data. If confirmed, minimum inhibitory concentrations that exceeded alert criteria were promptly reported to the Ministry of Health and to sentinel clinics.

Disk diffusion for isolates for all antibiotics, with the exception of cefoxitin, were reported. Etests were used for an initial subset of 116 isolates as well as for some later specimens (if Etest strips were available) or when isolates met minimum inhibitory concentration alert value criteria. Data on cefoxitin were included in the comprehensive analysis of 116 samples using Etest. *P* values ≤.05 were deemed statistically significant. Data were compared using two-tailed independent *t* tests with unequal variance for continuous variables and using the chi-square test for categorical variables. Odds ratios (OR) and their associated confidence intervals were calculated using logistic regression. All analyses were performed using SAS statistical software (version 9.4; SAS Institute Inc).

### Ethical Approval

The gonococcal surveillance program was performed as part of public health surveillance activities under the purview of the Ugandan Ministry of Health and approved by the Director General. As the activity was permitted by the Ministry of Health in the interest of public health practice for disease surveillance in Uganda, it did not fall within the realm of research. The gonococcal surveillance program legitimately involved individuals who were not explicitly asked to provide informed consent and did not require further institutional review board approval.

## Results

### Overall Sample Characteristics

Results are presented for the overall sample characteristics and are further analyzed by gonorrhea culture–status.

Urethral samples (N=639) were collected from men whose mean age was 26.9 (SD 9.6) years, and of whom, 7.2% (46/639) self-reported HIV-positive (Table 1). Microscopic diagnosis of presumptive gonorrhea was positively made from evidence of Gram-negative intracellular diplococci from urethral material in 414/639 (64.8%) samples; evidence of Gram-negative intracellular diplococci was not present in 225/639 (35.2%) of samples. Only 400 (400/414, 96.6%; 400/639, 62.6%) Gram-negative intracellular diplococci–positive samples on Gram staining were culture-positive, based on the growth of typical small translucent colonies on modified Thayer-Martin medium, and of these 399 (399/414, 96.4%; 399/639, 62.4%) were oxidase- and superoxidase-positive consistent with gonorrhea. Overall, 36% of samples (230/639) were culture-negative. Of the culture-positive samples, 60.8% (243/400) were from individuals self-reported without HIV, 7.5% (30/400) were from individuals self-reported HIV-positive, and 31.8% (127/400) were from individuals whose HIV-status was unknown.

Reported condom-use was low; 2.7% (18/639) reported always using a condom and 69.3% (443/639) reported one or more sexual partners within the past 6-12 months (mean 1.4; SD 1.49; range 0-20). Full demographic, behavior, and health-related characteristics are presented in Table 1.

**Table 1:** Demographic, health-related, and behavior factors.

Variable		Samples (N=639), n (%)
**HIV-status**		
	Positive	46 (7.2)
	Negative	393 (61.5)
	Unknown	200 (31.3)
**Condom use^a^**		
	Always	18 (2.8)
	Sometimes	403 (63.1)
	Never	176 (27.5)
**Number of sex partners^b,c^**		
	0	189 (29.1)
	1	196 (31.0)
	2	146 (23.1)
	3	61 (9.7)
	4	12 (1.9)
	5	14 (2.2)
	6	7 (1.1)
	7	1 (0.2)
	8	1 (0.2)
	10	4 (0.6)
	20	1 (0.2)
**Symptoms**		
	Discharge	590 (88.2)
	Dysuria	553 (86.5)
	Other	36 (5.6)
**Previous history of gonorrhea**		
	Yes	286 (44.8)
	No	353 (55.2)
**Sex for money^b^**		
	Yes	98 (15.3)
	No	541 (84.7)
**Recent antibiotic use^d^**		
	Yes	209 (32.7)
	No	430 (67.3)

^a^n=42 responses were missing.

^b^in the past 6 or 12 months.

^c^n=7 responses were missing.

^d^in the past 14 or 60 days.

### Resistance Profiles

Table 2 and Table 3 show the resistance characteristics found by disk diffusion and by Etest, respectively. Fewer Etests were performed because of difficulty in obtaining test kits. Disk diffusion showed greater than 91% (364/399) resistance to penicillin and greater than 99% (397/399; 399/399) resistance to both tetracycline and ciprofloxacin. A resistance of 1.06% (2/188) to ceftriaxone and resistance or intermediate resistance of 2.1% (4/188) to azithromycin were found using Etest. Early samples (n=116) collected from September 2016 to March 2017 underwent comprehensive microbiological analysis and 59.5% of these (69/116) underwent complete Etest resistance profiles. Of these, 96% (66/69) were ciprofloxacin-resistant or ciprofloxacin-intermediate, no resistance (0/69) to cefixime was demonstrated, and 96% (66/69) of isolates were azithromycin- and gentamicin-sensitive (Table 4).

The minimum inhibitory concentrations needed for inhibiting 50% of microbial growth for ceftriaxone, cefixime, gentamicin, ciprofloxacin, and azithromycin were 0.003 µg/mL, 0.0016 µg/mL, 3 µg/mL, 2 µg/mL, and 0.19 µg/mL, respectively. Additionally, 2 isolates demonstrated high-level resistance to azithromycin by Etest (minimum inhibitory concentrations=12 µg/mL and 16 µg/mL) and 1 isolate demonstrated intermediate resistance to azithromycin (minimum inhibitory concentration=3 µg/mL); 3 isolates were gentamicin-intermediate (all 3 with minimum inhibitory concentration=6 µg/mL) and were sensitive to azithromycin.

**Table 2:** Disk diffusion resistance characteristics.

Antibiotics	Breakpoint in mm	ZOI^a^ range in mm	Resistant, n (%)	Intermediate, n (%)	Sensitive, n (%)
Cefixime (n=395)	S^b^≥31	28-60	0 (0)	1^c^ (0.25)	394 (99.7)
Ceftriaxone (n=397)	S≥35	28-62	2^d^ (0.5)	2^d^ (0.5)	393 (99.0)
Ciprofloxacin (n=399)	≥41	0-27	399 (100)	0 (0)	0 (0)
Penicillin G (n=399)	≥47	10-52	364 (91.2)	34 (8.5)	1 (0.25)
Spectinomycin (n=34)	≥18	18-34	0 (0)	1 (2.9)	33 (97.1)
Tetracycline (n=399)	≥38	6-40	397 (99.5)	1 (0.25)	1(0.25)

^a^ZOI: zone of inhibition.

^b^S: sensitive.

^c^ZOI of 28 mm.

^d^2 had ZOI of 28 mm, 1 had ZOI of 32 mm, and 1 had ZOI of 34 mm.

**Table 3:** Etest resistance characteristics.

Antibiotics		Resistant			Intermediate			Sensitive	
	Range (µg/mL)	n (%)	Breakpoint (µg/mL)	n (%)		Breakpoint (µg/mL)	n (%)		Breakpoint (µg/mL)
Azithromycin (n=188)	<0.016-16	2 (1.1)^a^	≥8	2 (1.1)^b^		—	184 (97.9)		≤2
Cefixime (n=185)	<0.016-0.16	0 (0)	—	—		—	185 (100)		≤0.25^c^
Ceftriaxone (n=188)	0.002-1.5	2 (1.1)^d^	—	—		—	186 (98.9)		≤0.25^c^
Ciprofloxacin (n=192)	0.002-32	188 (97.9)	≥1	—		—	4 (2.1)^e^		≤0.061
Gentamicin (n=189)	0.38-48	1 (0.5)^f^	≥32	3 (1.6)^g^		8-16	185 (97.9)		≤4

^a^Minimum inhibition concentrations (MIC) are noted here;1 sample had MIC=12 µg/mL; 1 sample had MIC=16 µg/mL.

^b^2 samples had MIC=3 µg/mL.

^c^2 samples had MIC=0.125 µg/mL.

^d^1 sample had MIC=1.5 µg/mL; 1 sample had MIC=0.5 µg/mL.

^e^2 samples had MIC=0.02 µg/mL; 1 sample had MIC=0.016 µg/mL; 1 sample had MIC=0.008 µg/mL.

^f^1 sample had MIC=48 µg/mL.

^g^3 samples had MIC=6 µg/mL.

**Table 4:** Percentage of isolates susceptible to the antibiotic tested.

Antibiotic	Disk diffusion	Etest (%)
Azithromycin	NT^a^	95.6
Cefixime	100	100
Ciprofloxacin	4.3	0.0
Ceftriaxone	99.0	97.0
Cefuroxime	100	90.0
Cefoxitin	84.0	NT
Gentamicin	25.0	95.7
Penicillin	0.0	NT
Spectinomycin	97.0	NT
Tetracycline	0.0	NT

^a^NT: not tested.

The gonorrhea samples (4/400, 1% of culture-positive) that demonstrated decreased susceptibility to ceftriaxone by disk diffusion were different by Etest where resistance was set at ≥0.25 µg/mL. In the 5 isolates with results by both methods, 2 were intermediate, 1 was resistant, and 2 were sensitive by disk diffusion, compared with 2 resistant and 3 sensitive by Etest; full details are in Multimedia Appendix 2. The 2 (0.5%) gonorrhea isolates demonstrating intermediate or decreased minimum inhibitory concentration to cefixime by disk diffusion were found to be sensitive by Etest, when that test was performed, where resistance was set at ≥0.25 µg/mL (Multimedia Appendix 2). There were 4/188 (2.1%) gonorrhea isolates that demonstrated intermediate or decreased minimum inhibitory concentration to azithromycin by Etest where sensitivity was set at ≤2.0 µg/mL (Multimedia Appendix 2).

Table 5 categorizes the data by gonorrhea culture–status. Men with HIV were significantly older (*P*<.001). Self-reported HIV positivity of 7.2% was higher than the national 5.3% (range 5.0%-5.7%) in men aged 15 to 49 years and substantially higher than the 1.9% reported in those aged 15 to 24 years reported by Joint United Nations Program on HIV/AIDS [25]. Rates of condom use were significantly different with men with HIV more likely to report always using a condom (*P*<.001); these men were more likely to have accessed antibiotics prior to their clinic visit for urethritis (*P*=.006; data not shown). Men whose samples were gonorrhea culture–positive were younger (*P*=.01) and less likely to always use condoms (*P*=.003). Men who reported sometimes using condoms were more likely to be found *N gonorrhoeae* culture–positive compared to those who never used condoms (Odds ratio [OR] 1.77, 95% CI 1.23, 2.55). Men whose samples were *N gonorrhoeae* culture–positive had fewer numbers of previous *N gonorrhoeae* episodes than those whose samples were culture-negative (*P*<.001); for every previous episode they had a 0.68 odds of being *N gonorrhoeae* culture–positive. Only 43.3% (173/400) of men whose samples were culture-positive had been prescribed the recommended treatment of an extended-spectrum cephalosporin and doxycycline as syndromic management of urethral discharge syndrome [26].

**Table 5:** Variables by gonorrhea culture–status.

Variable		All^a^, N=639	Negative, n=230	Positive, n=400	Odds Ratio (95% CI)	*P* value
Age, mean (SD)		26.9 (9.6)	28.3 (10.2)	26.3 (9.0)	0.98 (0.96, 0.99)	.01
**HIV-status, n (%)**						.67
	Positive	46 (7.2)	16 (7.4)	30 (7.8)	1.13 (0.60, 2.15)	
	Negative	393 (61.5)	147 (68.1)	243 (64.5)	REF	
	Unknown	200 (31.3)	67 (29.1)	127 (31.8)	1.147 (0.80, 1.64)	
**Engage in commercial sex, n (%)**						.13
	Yes	98 (15.3)	42 (18.3)	55 (13.8)	0.71 (0.46, 1.11)	
	No	541 (84.7)	188 (81.7)	345 (86.3)	REF	
**Condom use^b^, n (%)**						.003
	Always	18 (3.0)	8 (3.8)	7 (1.9)	0.71 (0.25, 2.05)	
	Sometimes	403 (67.5)	126 (59.4)	274 (72.7)	1.77 (1.23, 2.55)	
	Never	176 (29.5)	78 (36.8)	96 (25.5)	REF	
**Previous episodes of gonorrhea^c^, n**					0.68 (0.56, 0.83)	<.001
	mean (SD)	0.43 (0.87)	0.62 (1.13)	0.32 (0.66)	N/A	
	range	0-8	0-8	0-3	N/A	
Number of sex partners^d^, mean (SD)		1.4 (1.7)	1.4 (2.0)	1.4 (1.5)	0.99 (0.91, 1.10)	.98
**Recent antibiotic use, n (%)**					0.73 (0.52, 1.02)	.07
	Yes	209 (32.7)	86 (37.4)	121 (30.3)	N/A	
	No	430 (67.3)	144 (62.6)	279 (69.8)	N/A	
**Antibiotic therapy^e^, n (%)**						.17
	Correct combination	254 (42.3)	85 (39.7)	173 (43.3)	REF	
	Correct combination plus metronidazole or tinidazole	199 (33.2)	65 (30.4)	132 (35.0)	0.94 (0.64, 1.40)	
	Overtreatment	42 (7.0)	19 (8.9)	23 (6.1)	1.58 (0.82, 3.07)	
	Incorrect antibiotic combination	106 (17.5)	45 (21.0)	59 (15.7)	1.46 (0.92, 2.34)	

^a^n=9 missing culture-status.

^b^n=42 responses were missing.

^c^n=21 responses were missing.

^d^n=7 responses were missing.

^e^n=38 responses were missing.

## Discussion

### Principal Findings

The WHO STI guidelines currently recommend that reliable and recent local resistance data should guide the choice of either single or dual therapy for genital and anorectal gonococcal infection [27]. While agar dilution is considered the gold standard for determining antimicrobial susceptibility of *N gonorrhoeae* [28], it is labor-intensive and requires technical expertise that is often not available in resource-limited settings. Disk diffusion is widely used in microbiology laboratories for antibiotic-sensitivity testing, but reproducibility is a significant issue. While Etests are more reliable, simpler, and faster to perform, and its results have demonstrated acceptable agreement with those from agar dilution [29,30], the cost of Etests may be an issue in resource-limited settings. Our data demonstrate good concordance between resistance measured by disk diffusion and that measured by Etest for most antibiotics that were tested (with the exception of gentamicin). For other antibiotics, where comparison between methods was possible, discordance ranged from 0% to 10%. The reason for the 71% difference between the two different sensitivity-testing methods for gentamicin is not known and requires further investigation. Gentamicin’s Etest results are most likely to be correct; similar, though less pronounced differences by assay type have been reported [31].

In Uganda, between 1993 and 2010, ciprofloxacin was the recommended first-line antibiotic for presumptive gonorrhea. Since 2012, Ugandan Clinical Guidelines recommend a single dose of cefixime with doxycycline taken over 7 days for syndromic management of urethral discharge syndrome [26]. The antimicrobial resistance profiles reported here have profound public health implications; they support arguments for expanded surveillance programs and investment in first-line antibiotic supplies which could improve population health and slow the spread of antimicrobial-resistant gonorrhea in Uganda. In 2018, the annual cost of EGASP in Kampala was approximately 247,000,000 UGX (US $64,996; an exchange of 1 UGX=US $0.0026 was applied). Of the total, 23% of costs were for antimicrobial testing, 4% for clinic staff, and 17% for the sites.

Recently, ceftriaxone-resistant gonorrhea strains have been found in Asia, Europe, and North America [8,11,32]. High-level azithromycin resistance has also been reported [7,10]. This marks a low point in the battle against antimicrobial-resistant gonorrhea since both ceftriaxone and azithromycin are recommended by the Centers for Disease Control and Prevention and other agencies as first-line therapies [27,33]. As a result, the Centers for Disease Control and Prevention and WHO have called for the strengthening of global surveillance [20]; in Africa, surveillance has been inadequate or virtually absent [34]. The emergence of gonorrhea with decreased sensitivity to current first-line antibiotics raises the specter of untreatable multidrug resistance. *N gonorrhoeae* has evolved to outpace every new class of antibiotic that has been routinely used in its treatment. The pipeline for new antigonococcal therapies is narrow with no readily available, affordable, and orally administered drugs close to being accessible for routine clinical use [5]. New drugs such as the novel oral fluoroketolide or solithromycin show potential but are not FDA-approved, and the recycling of older drugs, such as gentamicin, is limited by potential toxicity and less-than-ideal effectiveness (<92% microbiological cure). Even carbapenems are not guaranteed to have enduring efficacy since extended-spectrum cephalosporin resistance determinants (for example, mosaic *penA* alleles, *mtrR*, and *penB*) also increase the ertapenem minimum inhibitory concentration [12].

Antimicrobial resistance can readily develop in sub-Saharan Africa because of limited surveillance and poor antimicrobial stewardship. The feasibility of undertaking gonorrhea surveillance in a resource-limited setting on a relatively large scale has been established [35]. It is likely that urethral discharge syndrome represents a small portion of actual antibiotic resistance since cervical, rectal, and pharyngeal infections are often asymptomatic [36,37].

These data reveal a population with higher than national average HIV prevalence (7.2% versus 5.3%), substantial rates of transactional sex (15.3%), a high number of partners, and very low consistent condom-use (less than 3.0%). Those who reported sometimes using condoms in comparison to those who reported never using condoms were found to be *N gonorrhoeae* culture–positive more often. This counterintuitive finding may reflect that men who engaged in lower-risk sexual encounters with regular partners were less likely to use condoms but requires further exploration. Men with previous episodes of gonorrhea were found to be less likely to have a positive *N gonorrhoeae* culture. The explanation for this is not clear but may reflect differences in antibiotic use prior to attending clinic, or raises the possibility of unexplained, modest, inducible immune responses to repeated *N gonorrhoeae* infection [38]. Less than 50% received recommended first-line antibiotic treatment. Even in high-resource countries such as the United States, in 2016, approximately 20% of individuals received nonrecommended regimens for gonorrhea [39]. Ciprofloxacin use was common despite its almost universal resistance; indeed, ciprofloxacin antimicrobial resistance appears to have increased dramatically in the past decade from 83.1% in 2008 and 2009 [18] to 97.9% in this analysis (measured by Etest). Azithromycin resistance has increased from 2.7% in 2008 and 2009 [18] to 4% in this analysis. In 2008 and 2009, there was no documented cefixime resistance which should offer some reassurance, since cefixime is a WHO and Ugandan recommended first-line treatment; however, anecdotal evidence suggests that cefixime is generally unavailable in STI clinics, and that patients are unable to afford the drug. Cefixime’s continued potency may be the result of its own scarcity. Data on same-sex sexual activity were not collected, since disclosure could be potentially dangerous [24]. The Joint United Nations Program on HIV/AIDS reported a much higher rate of HIV in Ugandan men who have sex with men (13.2%) than that in men who have sex with women (5.3%) [25], so it is likely that *N gonorrhoeae* prevalence is also higher, particularly at extragenital sites. There were low rates of antimicrobial resistance to both gentamicin and extended-spectrum cephalosporins, but they were measurable so, nevertheless, require monitoring. In contrast, there was no recorded gentamicin resistance in Malawi in 2007 [31]. The finding of isolates (4/188, 2.1%) with increased minimum inhibitory concentration to azithromycin was concerning since, in many regions, azithromycin is a common component of dual *N gonorrhoeae* therapy [33], and Uganda may be compelled to follow suit given the extremely high rates of doxycycline resistance.

There was overtreatment in up to 11.6% of men which included oral cefixime and parenteral ceftriaxone in the same individual. Only 414/639 (64.8%) of men presenting with discharge had Gram-negative intracellular diplococci on Gram stain or a positive gonorrhea culture, compared with 73.5% in an earlier Zimbabwean study [40] that used multiplex polymerase chain reaction which is more sensitive than culture. The remaining 35.2% had, by definition, nongonococcal urethritis and were likely to have other unrecognized STIs.

### Limitations

The study was limited by several factors including the lack of biochemical or molecular testing to confirm nutrient requirements and that typical morphologies and oxidase reactions represented *N gonorrhoeae* rather than related *Neisseria* species. In addition, the data on previous gonorrhea- and HIV-status were collected by self-report and 31.3% of the men were not aware of, or did not report, their HIV-status. There was no clinical outcome data, and the nature of antibiotic treatment taken prior to clinic attendance was unknown. Of the 414 samples with Gram-negative intracellular diplococci on microscopy, 400 (96.6%) were *N gonorrhoeae* culture–positive while the remainder resulted in nonsignificant growth which could represent culture failure or the presence of other morphologically similar species. Firm conclusions about concordance between antibiotic sensitivity methods in the samples that demonstrated intermediate or decreased minimum inhibitory concentration to azithromycin is difficult because of incomplete testing by both methods.

We had no data on same-sex activity or extragenital exposures nor did we have data on the prevalence of other STIs. Finally, different data collection or case report forms were used; therefore, some of the data points were collected across different periods (over 6 months versus 12 months) which may have influenced recall bias.

### Conclusion

Data on *N gonorrhoeae* resistance in sub-Saharan Africa have been lacking to date, with the exception of sporadic studies and reports [21,22,41], and more recently, increasing systematic efforts [35]. This study has clearly demonstrated resistance to and increased minimum inhibitory concentration for vital antigonococcal antibiotics. It supports the hypothesis that several antibiotics are obsolete for use in the treatment of gonorrhea in Uganda. Ciprofloxacin resistance is much higher in Uganda than described elsewhere despite its removal from treatment guidelines more than 5 years ago—resistance to it has persisted and ciprofloxacin cannot be recycled. Measurable azithromycin and gentamicin resistance and decreased sensitivity as well as increasing minimum inhibitory concentration for ceftriaxone are worrisome. With selection pressure being exerted by the frequent use of extended-spectrum cephalosporins for numerous febrile illnesses in Africa, it may not be long before these strains are endemic in Uganda. There were very high rates of sexual risk in this population and there is a need for prevention interventions such as HIV pre-exposure prophylaxis. Antimicrobial stewardship programs in conjunction with continued surveillance will be critical in preventing the expansion of antimicrobial-resistant gonorrhea and will be important for filling gaps in knowledge, identifying emerging trends, and creating data-driven action plans.

## Appendix

Multimedia Appendix 1 Ugandan National treatment guidelines.

Multimedia Appendix 2 Additional results.

## Abbreviations

EGASP: Enhanced Gonococcal Antimicrobial Surveillance Program
FDA: Food and Drug Administration
GASP: Gonococcal Antimicrobial Surveillance Program
GNID: gram-negative intracellular diplococci
HIV: Human Immunodeficiency Virus
MIC: minimum inhibitory concentration
OR: odds ratio
STI: sexually transmitted infection
UGX: Ugandan shilling
WHO: World Health Organization
ZOI: zone of inhibition
