# Supplementary material for: Antimicrobial Resistance of Neisseria Gonorrhoeae in a Newly Implemented Surveillance Program in Uganda: Surveillance Report
Source: JMIR Public Health Surveill. 2020 Jun 10;6(2):e17009. doi: 10.2196/17009 (PMC7315362; doi:10.2196/17009)
Supplement: Multimedia Appendix 1 [file publichealth_v6i2e17009_app1.docx]

Multimedia Appendix 1: Ugandan National treatment guidelines

Link to the Ugandan National treatment guidelines. Chapter 3 is on STI. <https://www.health.go.ug/sites/default/files/Uganda%20Clinical%20Guidelines%202016_FINAL.pdf>


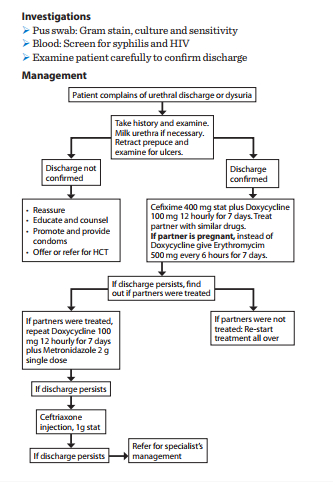


The syndromic approach to male urethral discharge syndrome in the Ugandan treatment guidelines. Page 270.
