# Supplementary material for: Antimicrobial Resistance of Neisseria Gonorrhoeae in a Newly Implemented Surveillance Program in Uganda: Surveillance Report
Source: JMIR Public Health Surveill. 2020 Jun 10;6(2):e17009. doi: 10.2196/17009 (PMC7315362; doi:10.2196/17009)
Supplement: Multimedia Appendix 2 [file publichealth_v6i2e17009_app2.docx]

Multimedia Appendix 2: Additional results.

| ID | Age | Previous gonorrhea history | Recent Antibiotic exposure | Antibiotic treatment dispensed | Antibiotic sensitivity testing | Ciprofloxacin | Gentamicin | Penicillin G | Tetracycline | Cefixime  (ZOI mm) | **Ceftriaxone**  (ZOI mm) | Azithromycin |
| --- | --- | --- | --- | --- | --- | --- | --- | --- | --- | --- | --- | --- |
| 37 | 28 | N | Y | Cefixime , Doxycycline | disk diffusion | R | ND | R | R | ND | **I (34)** | ND |
|  |  |  |  |  | Etest® | 2.0 | 4.0 | ND | ND | 0.016 | 0.064 | 0.19 |
| 59 | 52 | Y | Y | Cefixime , Doxycycline | disk diffusion | R | R | R | R | ND | **R (28)** | ND |
|  |  |  |  |  | Etest® | 4.0 | 4.0 | ND | ND | 0.125 | 0.125 | 0.5 |
| 124 | 21 | N | N | Ceftriaxone , Doxycycline | disk diffusion | ND | ND | ND | ND | S (32) | **R (28)** | ND |
|  |  |  |  |  | Etest® | ND | ND | ND | ND | ND | ND | ND |
| 130 | 36 | Y | N | Cefixime , Ceftriaxone , Doxycycline | disk diffusion | R | ND | R | R | S (34) | S (35) | ND |
|  |  |  |  |  | Etest® | 3.0 | 4.0 | ND | ND | 0.016 | **1.5** | 0.19 |
| 465 | 20 | N | N | Cefixime , Doxycycline, Metronidazole | disk diffusion | R | ND | R | R | S (40) | **I (32)** | ND |
|  |  |  |  |  | Etest® | 1.0 | 1.0 | ND | ND | <0.016 | <0.016 | 0.25 |
| 474 | 42 | Y | N | Ceftriaxone , Doxycycline | disk diffusion | R | R | R | R | S (38) | S (44) | ND |
|  |  |  |  |  | Etest® | 20 | 2.0 | ND | ND | <0.016 | **0.5** | 0.25 |

**Table 3: a. Isolates with different susceptibility to ceftriaxone by disk diffusion and by Etest**

I – intermediate, N – no, ND – not determined, S – sensitive, R – resistant, Y – yes, ZOI – zone of inhibition

**Table 3: b. Isolates with different susceptibility to cefixime by disk diffusion and by Etest**

| ID | Age | Previous gonorrhea history | Recent Antibiotic exposure | Antibiotic treatment dispensed | Antibiotic sensitivity testing | Ciprofloxacin (ZOI mm) | Gentamicin | Penicillin G | Tetracycline (ZOI mm) | **Cefixime**  (ZOI mm) | Ceftriaxone  (ZOI mm) | Azithromycin |
| --- | --- | --- | --- | --- | --- | --- | --- | --- | --- | --- | --- | --- |
| 214 | 20 | Y | Y | Cefixime | disk diffusion | R | ND | R | R | **S (31)** | S (36) | ND |
|  |  |  |  |  | Etest® | ND | ND | ND | ND | ND | ND | ND |
| 616 | 20 | Y | Y | Ciprofloxacin, Doxycycline, Metronidazole | disk diffusion | R (11) | ND | R | R (10) | **I (28)** | S (38) | ND |
|  |  |  |  |  | Etest® | 4.0 | 3.0 | ND | ND | 0.023 | 0.023 | 0.25 |

I – intermediate, N – no, ND – not determined, S – sensitive, R – resistant, Y – yes, ZOI – zone of inhibition

**Table 3: c. Isolates with different susceptibility to azithromycin by disk diffusion and by Etest**

| ID | Age | Previous gonorrhea history | Recent Antibiotic exposure | Antibiotic treatment dispensed | Antibiotic sensitivity testing | Ciprofloxacin | Gentamicin | Penicillin G | Tetracycline | Cefixime  (ZOI mm) | Ceftriaxone  (ZOI mm) | **Azithromycin** |
| --- | --- | --- | --- | --- | --- | --- | --- | --- | --- | --- | --- | --- |
| 3 | 47 | N | N | Ceftriaxone , Doxycycline | disk diffusion | R | ND | R | R | 35 (S) | 36 (S) | ND |
|  |  |  |  |  | Etest | 2.0 | 2.0 | ND | ND | 0.064 | 0.002 | **12.0** |
| 20 | 25 | Y | N | Ciprofloxacin, Doxycycline, Metronidazole | disk diffusion | R | ND | R | R | 35 (S) | 36(S) | ND |
|  |  |  |  |  | Etest | 3.0 | 2.0 | ND | ND | 0.016 | 0.006 | **16.0** |
| 102 | 24 | Y | Y | Ceftriaxone , Doxycycline | disk diffusion | R | ND | R | R | 47 (S) | 40 (S) | ND |
|  |  |  |  |  | Etest | 0.75 | 2.0 | ND | ND | 0.016 | 0.006 | **3.0** |
| 126 | 24 | N | N | Cefixime , Doxycycline, Metronidazole | disk diffusion | R | ND | R | R | 34 (S) | 38 (S) | ND |
|  |  |  |  |  | Etest | 3.0 | 48 | ND | ND | ND | ND | **3.0** |

I – intermediate, N – no, ND – not determined, S – sensitive, R – resistant, Y – yes, ZOI – zone of inhibition
